# Tooth Structure

**Published:** 1881-03

**Authors:** J. Taft


### THE DENTAL REGISTER.

VOL. XXXV.] MARCH, 1881. [No. 3.

### TOOTH STRUCTURE.

BY J. TAFT.

To understand correctly and fully any of the pathological states and conditions to which a vital organ or tissue may be subject, it is necessary to understand the nature of the structure, its anatomy, its formation, its physiology and the mode of its nutrition.

The bony tissues of the teeth consist of enamel, dentine and cement. The relation existing between them is very simple; the dentine constitutes the larger and inner part of the body and root, or roots, of every tooth. The enamel covers the crown completely, is thickest on the most prominent points, and terminates by an attenuated edge at the neck of the tooth. The root is covered by the cement; this is the thickest at and near the end of the root, and terminates by a thin edge at the neck of the tooth, and often overlaps the attenuated edge of the enamel, sometimes only meeting it, and in some cases even falling short of this.

The structural materials of the enamel are principally the hexagonal rods, or prisms, having upon their surface the merest trace or film of organic matter, and the enamel cuticle, or Nasmyth's membrane, which completely covers the external ends of the rods. This covering varies in thickness in different cases, and upon the masticating surfaces, at least, diminishes, with age and use. This is the hardest of all animal structures, and contains but the smallest trace of organic matter.

The length of the enamel rods corresponds to the depth of the enamel, less the thickness of the cuticle; they are straight and placed so that they are parallel with a line from the centre of the crown of the tooth to its periphery. They are so adapted and fitted together that no appreciable space exists between them, the six sided form being such as to most perfectly secure this result. The inner ends of the rods rest upon the periphery of the dentine, or more correctly, upon that almost structureless mass overlying the surface of the dentine, denominated the *granular layer*. Contour lines are often seen in the enamel, but they are not to be regarded as in any respect pertaining to or modifying the structure of the tissue, they are evidence of perturbation of nutrition during the period of development of the organ.

The granular layer, or strata, varies much in thickness and appearance, sometimes consisting of a mere film, at others presenting considerable thickness; in some cases it is totally structurless, in others it will have fibres often larger than the tubuli of the dentine, lying horizontally, or at right angles with the tubuli on the surface of the dentine. These sometimes interlace and twine together, and in other specimens will seem to run or lie independently of each other.

This layer varies greatly in density, sometimes being quite compact, and again it is quite porous, and lacking in a marked degree coherence. Of this layer Mr. Magitot says: "We cannot compare them to ossious corpuscles, but that they should rather be regarded as minute pits sunk in the thickness of the ivory at its exterior limit to aid the communications between the tubules which permeate this tissue. Therefore we have proposed to call this granular layer the *anastomatic plxus* of the dental (tubules) canaliculæ, meant to permit a free course to the fluid of imbibition, and thus to favor the organic movement."

The dentine, which constitutes the body of the tooth, presents a higher type of organization and structure than the enamel. It consists of tubes extending through the dentine from the centre to the periphery, the general course being about the same as that of the enamel rods, although exhibiting far less uniformity in this respect than these. The tubuli very rarely are straight; they show great variation in curvature, sometimes there will be a single curve embracing the entire length of the tube, and on the other hand a considerable number of curves will appear. To such an extent does this occur that the ordinary diagrams designed to show the microscopic appearance of dentine give a wave-like appearance to the tubuli. It should be kept in mind that there is great variety in this respect.

Sometimes the tubuli extend from one termination to the other, or from the pulp chamber to the periphery of the dentine without branching or anastomosis, but again there will be numerous branching; when, however, these are very numerous they are quite short and seem to terminate in sharp points. I have seen a a few specimens in which there seemed to be anastomosis of many of the tubes, but this is not often found. Where these are the larger branchings, it occurs on the outer half of the tube, these branches in some instances extend to the periphery of the dentine, and in others terminate before reaching it. The tubes are larger at the entrance to the pulp chamber than elsewhere, and some times diminish gradually from that point throughout the whole length. The peripheral terminations present considerable variety, in this the following will be found, viz :

First, termination at or near the surface of the dentine by an attenuated sharp point.

Second, ending at or near the surface without any contraction.

Third, the terminal ends sometimes expand and present a club-like appearance, and to such an extent is this sometimes found that the terminus may properly be said to be in cavities rather than in a club-like expansion.

Fourth, in a few cases they present a curved form extending to the formation of a definite loop.

How this loop-form of the ends of the tubuli is produced in the development of the tooth is not a matter of easy solution.

The tubules with all the branchings have walls or linings, consisting for the most part of organic matter, and varying in thickness in different cases, and at different points of the same tube. These canals contain fluid which has in solution, the nutrient supply for both the organic and inorganic constituents of the tooth. Hanover says: During life the canaliculæ inclose a transparent colorless fluid, containing calcarous matter in solution. This it doubtless does, and more, for the organic constituents require supply as well as the inorganic.

This nutrient supply is carried throughout the length of the tubes and their branchings, and most probably reaches the enamel.

For the clear illucidation of the manner of movement, and the change of this fluid in the tubes, demonstrative experiment has done very little. But from the changes that are constantly going on in living dentine, it can hardly be denied that movement and change of the contents of the tubuli do take place; on this point Mr. Magitot says: "As for ourselves, we are much disposed to believe that this liquid, charged with materials of untrition, bathes the canaliculæ through their whole extent, and accomplishes the double movement of assimilation and dis-assimilation thus setting up a certain exchange, slow indeed, but aided by the very disposition of the canaliculæ and thin multitudinous anastomosis."

The spaces between the tubes and their branches—the interstitial territory—are occupied chiefly by inorganic material, only small traces of organic matter are found in it; the amount of inorganic material is determined by the number and size of the tubuli and the branchings; usually the amount of interstitial matter decreases from the periphery to the central part of the tooth; this is due to the fact of the larger diameter of the tubes at and near their entrance to the pulp chamber, and there is less territory here than further out for their occupancy. The solidity and density of the dentine depends largely if not wholly upon the interstitial matter. It forms the walls of the tubuli and constitutes a solid mass outside of them.

*The cement*, that tissue which completely invests the dentine of the roots of the teeth, differs very materially from enamel and dentine. In a structural aspect it is a mean between dentine and true bone; it is far less dense than dentine, and wholly unlike it in structure. Its outer surface is rough, and to this is attached the periostium. It is thickest at the ends of the roots and at the other extreme terminates in a thin edge at the neck of the tooth usually a little over-lapping the thin edge of the enamel.

Lacunæ with many little canals extending out from each one, are liberally distributed through the cement, greatest in number in the thickest part, and becoming less as the tissue becomes thinner, till in the most attenuated part they are seldom found.

The lacunæ are sometimes in contact, or meet each other forming a comparatively large and elongated cavity. The canals from these cavities very commonly anastomose or run together so that a free communication is established through the structure. There are no Haversian canals in this structure, and red blood globules do not enter it. The cement is the medium of union between the dentine and the adjacent structures.

